# Study of Modified Magnesium Phosphate Cement for Fluoride Removal

**DOI:** 10.3390/ma16175749

**Published:** 2023-08-22

**Authors:** Sana Gharsallah, Abdulrahman Mallah, Abdulrahman Alsawi, Bechir Hammami, Mohamed Khitouni, Clarence Charnay, Mahmoud Chemingui

**Affiliations:** 1 Laboratory of Inorganic Chemistry, LR17-ES-07, Faculty of Science, University of Sfax, Sfax 3018, Tunisia; sana.gharsallah.etud@fss.usf.tn (S.G.); mahmoud.chmingui@fss.usf.tn (M.C.); 2 Department of Chemistry, College of Science, Qassim University, Buraidah 51452, Saudi Arabia; a.mallah@qu.edu.sa (A.M.); b.hammami@qu.edu.sa (B.H.); 3 Department of Physics, College of Science, Qassim University, Buraidah 51452, Saudi Arabia; ansaoy@qu.edu.sa; 4 Charles Gerhard Institut, UMR-5253 CNRS-UM-ENSCM, University of Montpellier, Place E, Bataillon, CEDEX 5, F-34095 Montpellier, France; clarence.charnay@umontpellier.fr

**Keywords:** magnesium phosphate cement (MPC), aluminum, fluoride adsorption, isotherms

## Abstract

In this study, we used a novel composite material based on magnesium phosphate cement (MPC) to explore the retention of fluoride from used water. Dead-burned magnesium oxide (MgO), ammonium dihydrogen phosphate (NH_4_H_2_PO_4_), and a few retarders were used to create this particular substance. Several studies have corroborated the performance of using aluminum in the capture of fluoride. From this perspective, we attempted to reinforce our matrix with different quantities of aluminum, which increased the resistance of the composite in water. The optimal conditions that were obtained were evaluated and scrutinized using a range of techniques, including scanning electron microscopy (SEM), X-ray diffraction (XRD), thermogravimetric analysis (TGA), Fourier transforms infrared spectroscopy (FTIR), and Brunauer–Emmett–Teller (BET). The adsorbents demonstrated a powerful ability to remove fluoride from contaminated water and the defluoridation capacity was evaluated at 4.84 mg/g. Equilibrium modeling was carried out, and the experimental data were expressed in accordance with the Langmuir, Freundlich, Temkin, and Dubinin–Radushkevich isotherms.

## 1. Introduction

Fluorine stands for the most electronegative element. Difluor is too reactive for direct use in its pure state. For this reason, it is always found in a reduced form as fluoride or in various combined forms such as fluorspar (CaF_2_), soloist (MgF_2_), fluorapatite (3Ca_3_(PO_4_)_2_ CaF_2_), and cryolite (Na_3_AlF_6_) [1,2]. In different parts of the world, fluoride is significant as a considerable natural contaminant in drinking water [3]. The use of fluoride is varied such as in industry and agriculture. Despite being naturally present in water, air, plants, and animals, fluoride is regularly supplemented to drinking water at a concentration of around 1 mg/L to effectively prevent tooth decay [4,5]. Nonetheless, the inherent issue arises when the fluoride concentration surpasses 1.5 mg/L [5], leading to the development of fluorosis, a condition that predominantly impacts teeth and bones, causing bone brittleness. Likewise, it leads to liver damage and causes endocrine glands, thyroid, and brain damage, Alzheimer’s syndrome, cancer, and infertility [2,6]. Numerous methods have been reported for the removal of fluoride from water, including nanofiltration, adsorption, reverse osmosis [7], membrane separation, and electrocoagulation [4]. The best technique used for this treatment is adsorption because it offers a moderate cost as well as great flexibility in the design and in the descaling operations [8]. The most often used adsorbents for the retention of fluoride from water are polymeric resins, activated carbon, activated alumina, metal oxides [7], chitosan, and biomaterials. Moreover, numerous studies confirmed the good performance of using aluminum in terms of capturing fluoride, which positively demonstrates its goodness of fit [9]. Yet, their main drawback resides in the fact that they are irrecoverable after adsorption. In this respect, we opted for a composite material that can be alloyed with these two compounds while maintaining their performance and recuperating aluminum after the adsorption of pollutants. These materials exhibit a strong attraction to fluorine, which enhances their ability to adsorb and retain it to a significant degree [2,8,10]. This composite material is based on magnesium phosphate cement, which represents a new type of binder formed at room temperature as a matrix that can retain it and enhance the adsorption in view of the presence of phosphate ion, and magnesium has a beneficial impact on the retention of fluoride [11].

Magnesium phosphate types of cement (MPC) are chemical cement belonging to the family of phosphate binder cement. The chemical reaction occurs between basic magnesia and an acid solution containing phosphates and, during this process, various components can be incorporated [12]. Struvite is indeed promising as a candidate compound for conditioning liquid radioactive waste from nuclear power plants [13]. The formation of this type of cement is too fast and very exothermic [14]. The equation of the reaction is written in the form:

MgO + NH_4_H_2_PO_4_ + 5 H_2_O    →   MgNH_4_PO_4_.6 H_2_O (struvite) (1)

With a chemically stable and porous structure, the cementitious matrix improves the adsorption and fixation of heavy metals [4,14]. The characteristics of the cement are notably influenced by multiple factors, including the reactivity of magnesia, the initial phosphate concentration, the magnesium-to-phosphate molar ratio (Mg/P), the use of set retarders, and the quantity of water added [12,15,16]. This type of cement has brought the greatest interest on account of its outstanding properties, namely low permeability, fast setting time, rapid hydration process, accelerated strength development, excellent adhesion with all surfaces, high resistance to an early stage, and fire-resistant properties and, furthermore, reduced drying and shrinkage [17,18]. Magnesium phosphate cement presents a broad range of applications, such as the rehabilitation of damaged civil structures and the swift restoration of deteriorated decks, highways, and airport runways. Moreover, it finds use in the design of biomedical materials for bone restoration, solidification, and stabilization of radioactive waste and heavy metal ions; additionally, it is employed in the stabilization of toxic materials and nuclear waste [19,20]. However, the main limitation of this type of cement is its reduced stability in water [21,22].

In the present study, we prepared a new composite material using magnesium phosphate cement as a matrix and introduced different amounts of aluminum to evaluate their ability to adsorb fluoride by examining their properties and modeling them according to different isotherms.

## 2. Materials and Methods

### 2.1. Materials

MPC pastes were prepared by blending various powders, including magnesia (magnesium oxide) (MgO > 99%,Merck KGaA Frankfurter Str.25, Darmstadt, Germany), ADP (NH_4_H_2_PO_4_ > 99%, Sigma-Aldrich (Darmstadt, Germany)), borax, which was used as a set retarder, and aluminum. All these materials were sourced from Sigma Aldrich. Magnesia was subjected to calcination at 1500 °C for 6 h to reduce its reactivity. Different quantities of renfort powder were added to the mixture. Aggregates were deliberately excluded from this research to avoid any additional interference from their impurities.

### 2.2. Preparation

The synthesis protocol involves combining specific quantities of magnesium and acid with the retarding agent, borax. We selected the suitable ratio, which is Mg/P = 1 with the required quantity of distilled water added. Subsequently, aluminum powder was incorporated in the appropriate quantity and was mixed until a homogeneous paste was obtained. The chemical compositions of different composites are depicted in Table 1.

### 2.3. Analysis Method

The microstructure of the cement was characterized through scanning electron microscopy (SEM, (JEOL)-Akishima, Japan) equipped with an energy dispersive spectrometer (EDS). The X-ray powder diffractometer X’per PRO PANalytical (Philips, Farnborough, UK) with CuKα radiation (λ_Kα_ = 1.54 Å) was used to determine the crystalline phases of the various samples. The diffraction patterns were obtained within the range of 10° < 2θ < 80°. Thermogravimetric analyses (TGA, Perkin-Elmer, Waltham, MA, USA) were performed to investigate the variations in sample mass as a function of temperature. The experiments were conducted in sweeping air over a temperature range of 20 to 1150 °C, using a heating rate of 20 °C/min^−1^. Fourier transform infrared spectrophotometer (FTIR, Perkin-Elmer, USA) was used in the range of 450–4000 cm^−1^ to determine the functional groups and the bonding patterns in different composites. Moreover, to assess the specific surface area and investigate the dehydration behavior of struvite, Brunauer, Emmett, and Teller (BET) analyses were carried out using the TriStar 3000 V6.06 A, UK.

## 3. Results

### 3.1. Composites Analysis

Despite its attractive and promising properties, the major problem with this type of cement lies in its lower resistance to water. As a matter of fact, we included merely a different amount of aluminum powder in order to enhance the water resistance of the cement; measures were taken to ensure its durability even in the presence of agitation over several days. In this regard, the samples with aluminum display the best properties and excellent resistance in the water. The analyses of different results reinforced the stability of the structure. Thus, we did not observe the formation of new phases, which put evidence that the added powders are inserted into the matrix. Furthermore, these materials could be the best choice to remove fluoride from waste water, since fluoride has a strong affinity with Al^3+^ and ${\mathrm{PO}}_{4}^{3-}$ ions.

**Figure 1:**
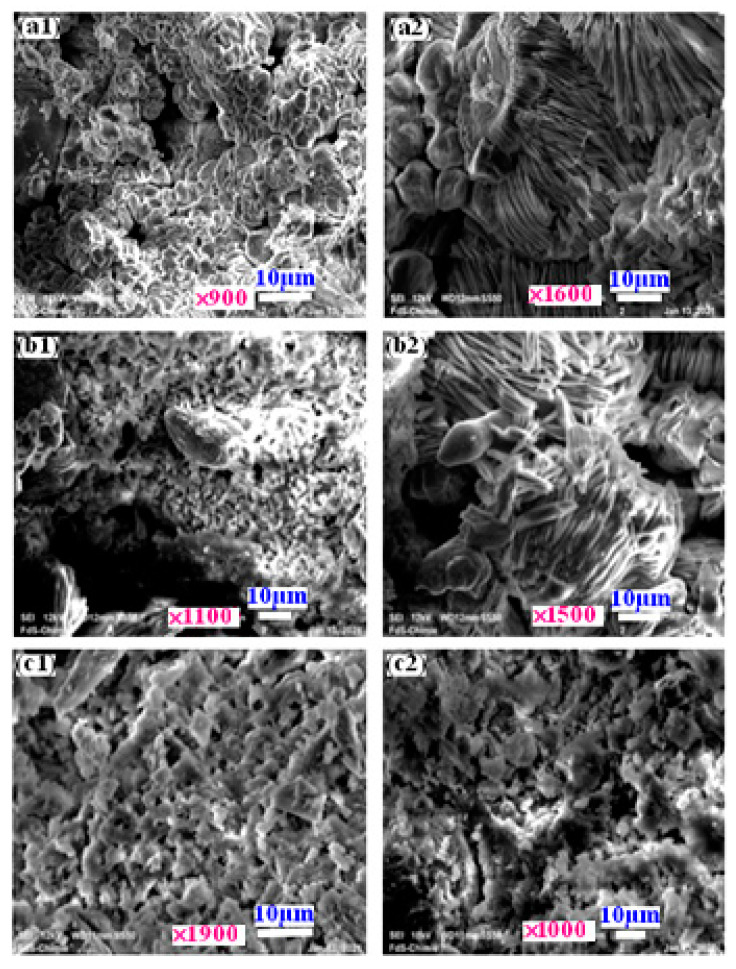
SEM images of composites samples S1 (**a1**,**a2**), S2 (**b1**,**b2**), and S3 (**c1**,**c2**).

SEM images of the new composites are given in Figure 1.

Indeed, Figure 1a1–c1 depict the microstructure of composites synthesized using different quantities of aluminum (S1, S2, and S3). These materials have more interesting properties. The microstructure of the sample appears compact and contains tubular crystals, generally in rod form. The overlapping of the crystals makes it possible to create a slight porosity, taking into account that the structure becomes harder; furthermore, the cement exhibits increased resistance to water contact, remaining stable and intact for over 15 days under continuous agitation. Moreover, the synthesis process resulted in a notable gas release, leading to a higher porosity rate.

**Figure 2:**
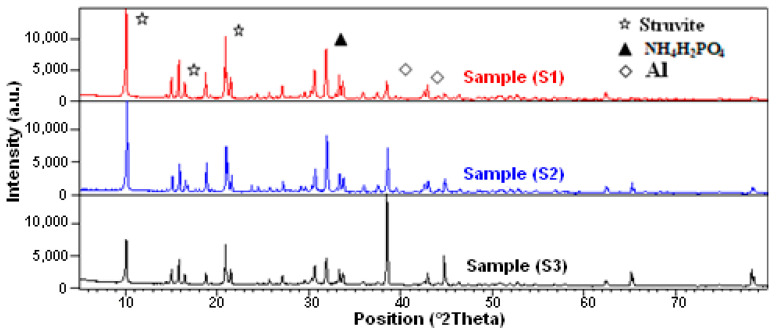
X-ray diffraction pattern of composites samples S1, S2, and S3.

The X-ray diffraction patterns of different synthesized composites S1, S2, and S3 are displayed in Figure 2.

Accordingly, one can observe the presence of the characteristic peaks of struvite and aluminum. Therefore, the material keeps its structure stable through adding aluminum. This is considered as an advantageous characteristic for this cement as it becomes harder and more water resistant. On the other side, no new phase is formed [23]. Moreover, one can observe the presence of a strong peak that is characteristic of struvite corresponding to the stable phase of cement in the position of 2θ equal to 21° and related to the crystallization of the material. Moreover, peaks relating to the residual NH_4_H_2_PO_4_ are observed in the interval of 2θ of 30–33° [12], while the principal peaks related to aluminum are in the range of 2θ of 38–39°. 

**Figure 3:**
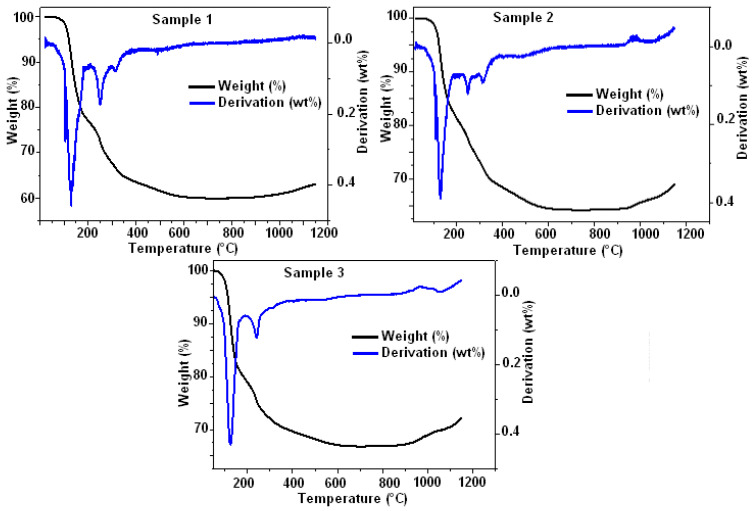
TGA-DTA curves of composites samples S1, S2, and S3.

Figure 3 shows the DTA-TG curves corresponding to the three composites S1, S2, and S3.

From the TGA curves, one can deduce that the decrease in weight starts at a temperature of 50 °C and ends at 700 °C. Moreover, the total mass loss calculated to be 40.07% can be divided into two parts related to the decomposition of the material. The residual mass remains until T = 1148.7 °C. From 50 to 300 °C, this decomposition corresponds to a dehydration reaction. The latter is written as follows:

MgNH_4_PO_4_.6 H_2_O    →   MgNH_4_PO_4_ + 6 H_2_O (g) (2)

In addition, from 300 to 700 °C, the decomposition reaction of the material is expressed in terms of:

MgNH_4_PO_4_    →   MgHPO_4_ + NH_3_ (g) (3)

For S1, S2, and S3, the losses in mass were similar. However, a slight gain in mass for different samples from 850 °C is noticed. This mass gain seems to be related to the oxidation of aluminum.

**Figure 4:**
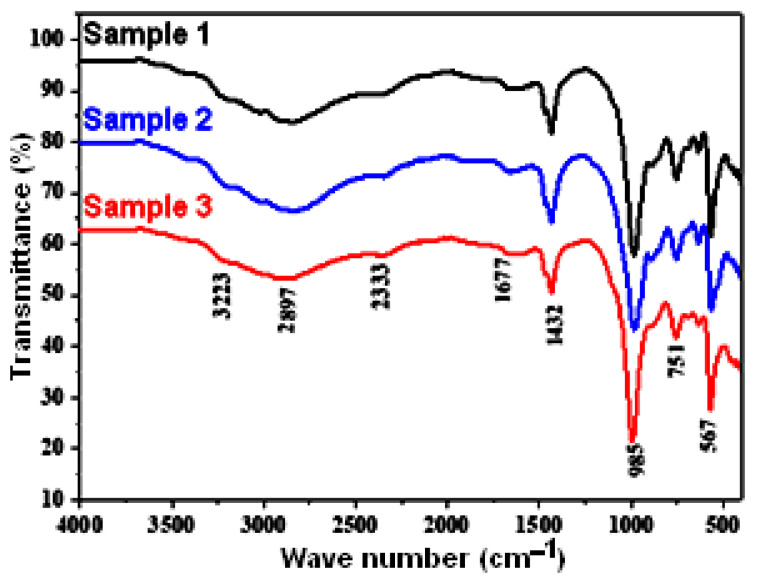
FTIR analysis of composite samples S1, S2, and S3.

The FTIR data of composite cement are displayed in Figure 4.

All samples exhibit almost the same bonds with slight shifts toward short- or long-wave numbers. The peak detected at approximately 3223 cm^−1^ provides confirmation of the existence of the –OH stretching bond of the chemisorbed water. The peak at 567 cm^−1^ may be attributed to the bending modes of P-O bonds in the phosphate groups. Compared to the previous work [4] (concerns a cement without aluminum addition), it is important to note that the introduction of aluminum powder in the cement composition allows the formation of the characteristic Al-O stretching vibrations observed in the range of 567 to 751 and, at 1677 cm^−1^, relates to the transient and stable phase of alumina (AlO_4_ or AlO_6_ vibrations). Adsorption bonds at 985 and at 2333 cm^−1^ are attributed to the P-O vibration asymmetry of ${\mathrm{PO}}_{4}^{3-}$ in the cement. The large peak at 2897 cm^−1^ is related to the unsymmetrical stretching vibration of the N–H in the ${\mathrm{NH}}_{4}^{+}$. The characteristic small peak of the unsymmetrical bending vibration of the ${\mathrm{NH}}_{4}^{+}$ group appears in the region of 1432 cm^−1^ [5,24,25].

**Figure 5:**
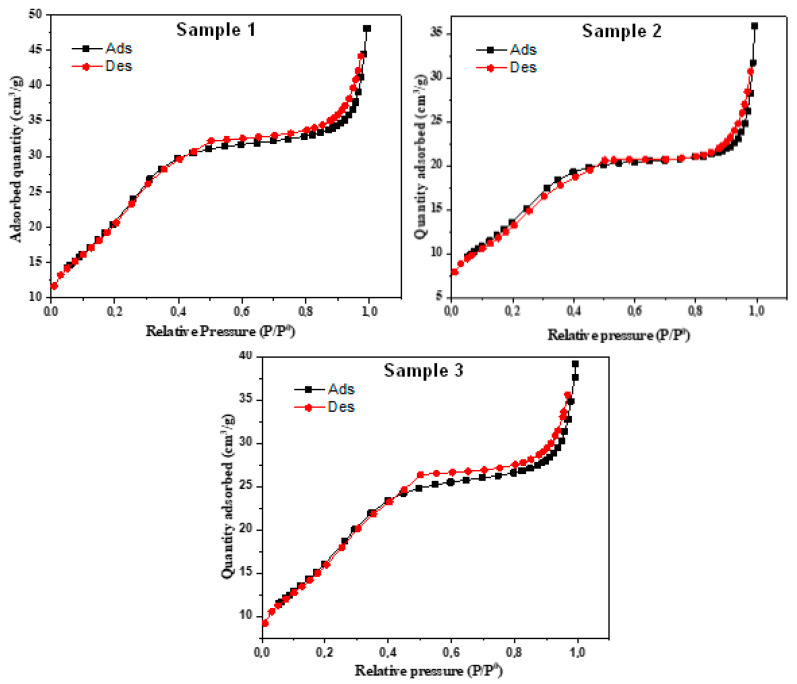
N2 adsorption/desorption curve for composites samples S1, S2, and S3.

The N2 adsorption/desorption curve presented in Figure 5 provides valuable insights into the porosity and surface characteristics of different types of cement.

The observed type II hysteresis curve, as commonly reported in BET analyses, indicates the presence of multilayer adsorption and adsorption on low-porosity surfaces, which typically initiate at higher pressures. The hysteresis loop, specifically of the H4 type, suggests that the sample contains split pores with a size distribution primarily in the microporous range. This finding implies the existence of fine pores within the cement structure, contributing to its overall porosity and potential for adsorption and desorption processes. Table 2 displays the crucial BET surface area, pore sizes, and volumes of the various cement types studied. These parameters provide essential quantitative data regarding the materials’ porosity and surface characteristics. The BET surface area signifies the extent of available surface sites for interactions, while the pore sizes and volumes offer insights into the specific pore structures and their capacities for adsorption. By correlating the information from Figure 5 and Table 2, it becomes evident that cement samples with larger BET surface areas tend to exhibit more significant porosity and, subsequently, enhanced adsorption potential. Moreover, the variations in pore sizes and volumes between different cement types highlight their unique pore architectures, which could influence their suitability for specific applications. Furthermore, the presence of split pores with a predominant microporous distribution in the H4 hysteresis loop suggests that the cement material possesses fine pores that might be particularly useful for the selective adsorption of certain molecules or ions.

### 3.2. Adsorption Study

**Figure 6:**
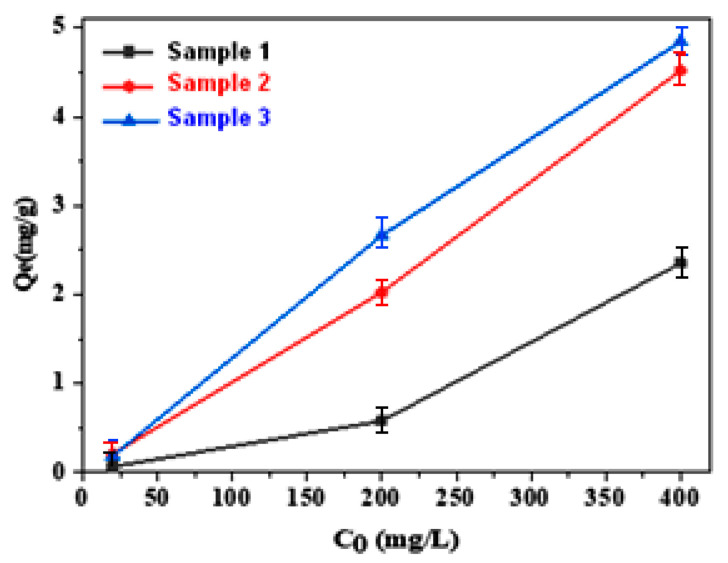
Influence of the initial concentration of fluoride on adsorption.

To explore the effect of different synthesized materials on the fluoride removal efficiency, these solids were examined with various concentrations. Fluoride removal increased with the increase in the initial concentration used and the experimental results are demonstrated in Figure 6.

Fluoride adsorption was carried out at room temperature (25 ± 2 °C) and a fixed pH for all samples. Using a constant solid mass of 0.3 g, 20 mL tubes containing fluoride solution with varying initial concentrations (from 20 to 400 mg/L) were stirred at a constant speed 200 rpm. The stirring process lasted overnight for 15 h. Then, they were filtered with 0.2 μm filters and the filtrate was then analyzed in terms of concentration of residual fluoride using a specific fluorine electrode. 

The quantity of fluoride adsorbed, represented as *q_e_* (mg/g), is determined using the following equation: [26,27]:

(4) $$q_{e}=\frac{\left(C_{0}-C_{e}\right)\times V}{m}$$

where *C*_0_ and *C_e_* are the initial and final concentrations of fluoride in solution (mg/L), *m* is the mass of adsorbent (g), and *V* is the volume of solution (L). 

**Figure 7:**
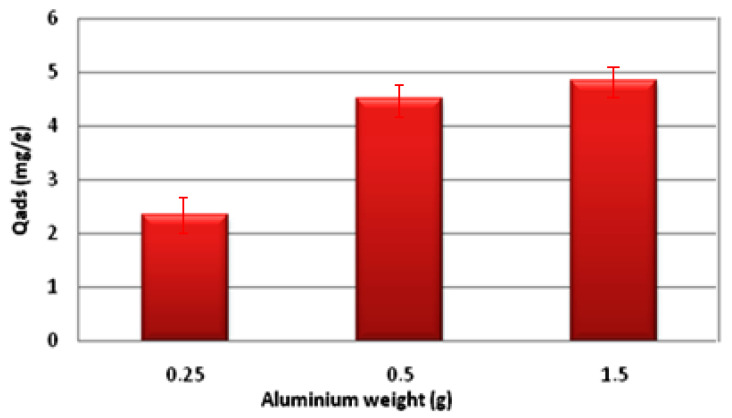
Effect of aluminum mass on adsorption long-wave.

The adsorption capacity of the various composite materials was tested. Figure 7 clearly demonstrates a positive correlation between the quantity of aluminum and the adsorption, indicating that an increase in aluminum leads to higher adsorption rates: from 2.35 mg/g for 0.25 g of Al (S1) to 4.51 mg/g for 0.5 g of Al (S2) and the optimal value is 4.84 mg/g for 1.5 g of Al (S3). Furthermore, the sorption of fluoride is predominantly influenced by the inclusion of aluminum, which exerts a positive impact on the material’s strength and hardness. Additionally, the presence of ${\mathrm{PO}}_{4}^{3-}$ ions also plays a role in the sorption process. 

The adsorption isotherms depict the relationship between the degree of adsorption and the concentration of the solute. The isotherm allows for determining the maximum quantity of adsorbed fluoride by giving a precise idea of the effectiveness of the various materials. Equilibrium studies were performed using different isotherms [9]. The Langmuir isothermal model indicates monolayer adsorption. The most used linearized form is [26]:

(5) $$\frac{1}{{\;q}_{e}\;}=\frac{1}{q_{m}}+\frac{1}{{\mathrm{bq}}_{m}C_{e}}$$

where q_m_ represents the maximum adsorption capacity in (mg/g), *b* is the Langmuir adsorption constant (L/mg), and the plots of $\frac{1}{{\;q}_{e}\;}=f\left(\frac{1}{C_{e}}\right)$ result in a straight line with a slope $\frac{1}{{\mathrm{bq}}_{m}}$ and an intercept $\;\frac{1}{q_{m}}$.

The Freundlich isotherm provides valuable insights into the efficiency of the adsorbent and the maximum adsorbate quantity that can be adsorbed. It represents a form of multi-layered adsorption, enhancing our comprehension of the adsorption process. The most used linearized form is [26]:

(6) $$\log q_{e}=\log K_{f}+\frac{1}{n}\;\log C_{e}$$

where the constant K_f_ (in mg/g) is an approximate indicator of the adsorption capacity, while n is a function of the strength of adsorption or heterogeneity factor. A plot of $\log q_{e}\;\mathrm{vs}.\;\log C_{e}$ results in a straight line with a slope $\frac{1}{n}$ and an intercept $\log K_{f}$.

The values of K_f_ and n are unique to a specific adsorbate–adsorbent system and can be used to identify and characterize it [25,26].

The Temkin isotherm introduces a factor (A) to gauge the impact of adsorbate–adsorbent interactions on the adsorption process. It is based on the concept that the heat of adsorption for all molecules in the adsorbed layer decreases linearly as the adsorbent surface coverage increases. The relationship is described by the following formula:

(7) $$q_{e}=B\;\ln(a\;C_{e})$$

linearized in the form [27]:

(8) $$q_{e}=\frac{\mathrm{RT}}{b}\;\ln A+\frac{\mathrm{RT}}{b}\;\ln C_{e}\;$$

where R is the gas constant (J/mol K), T is the temperature (K), b is the Temkin constant linked to the heat of sorption (j/mol), A is the Temkin’s isotherm constant (L/mg), and C_e_ is the equilibrium concentration (mg/L).

The Dubinin–Radushkevich isotherm showcases the adsorption’s multilayer nature and the involvement of Van der Waals forces. This model effectively elucidates the adsorption mechanism with Gaussian energy on heterogeneous surfaces. Notably, it finds a practical application for various ions in both physical and chemical adsorption [28,29]. The equation for the Dubinin–Radushkevich isotherm is provided below [30]:

(9) $$\ln q\;\varepsilon =\ln q_{m}-{\beta \;\varepsilon }^{2}$$

where ε is the Polanyi potential, $\varepsilon =\mathrm{RTln}\;\left(1+\frac{1}{C_{e}}\right)\;$, and β represents the adsorption energy.

To determine the suitability of various isothermal equations, the R2 regression coefficients were evaluated [31,32]. Based on the information presented in Table 3, it can be observed that all the samples adhere closely to the Langmuir model, which exhibits correlation coefficients closest to 1. This model is deemed most suitable for describing the adsorption behavior. Furthermore, the obtained value of the separation factor RL confirms that the adsorption process is favorable and relative to monolayer adsorption.

### 3.3. Comparison with Other Used Adsorbents

Comparing the defluoridation capacity of different adsorbents is fundamental to assess their relative performance. The comparison of the adsorption capacity of the materials synthesized with different adsorbents, with respect to their fluoride removal ability, is presented in Table 4. The Al-composite cement as a defluorinating agent proved to be better than multiple reported adsorbents.

**Figure 8:**
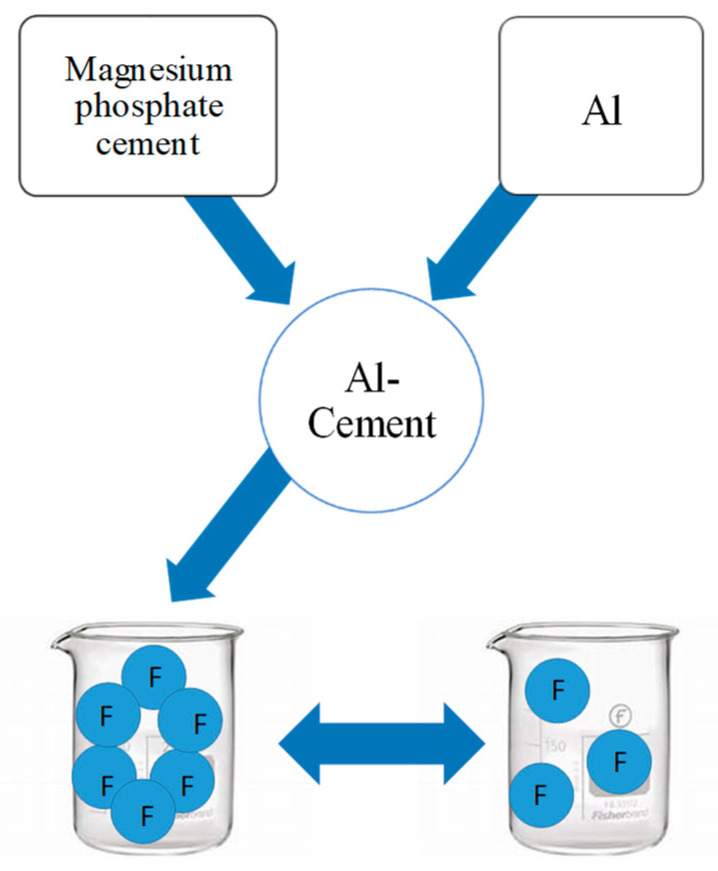
Schematic diagram for removing fluoride.

Based on all of these analyses and discussions, it is important to provide a visual representation to effectively understand the fluoride removal process. The schematic diagram proposed to remove fluoride has been given in Figure 8.

## 4. Conclusions

○ In the current research works, new composite material using magnesium phosphate cement was used as a matrix and different amounts of aluminum introduced in order to improve the resistance of cement in water and to use them in the adsorption of fluorine. We can conclude that the incorporation of different amounts of aluminum has a positive effect on the resistance of the material, which becomes harder and more resistant dry than in water, even after days. These materials have affinities for fluorine whose adsorption capacities we tested. The optimal adsorption values found are q_max_ = 4.84 mg/g. Moreover, the adsorption increases with the increase in the quantity of aluminum: from 2.35 mg/g for 0.25 g of Al to 4.84 mg/g for 1.5 g of Al. Furthermore, the Langmuir model was identified as the most appropriate fit for the synthesized materials, as it displayed the highest regression coefficients (R^2^ values closest to 1). Likewise, the observed isotherm corresponds to a monolayer adsorption process, and the calculated value of the separation factor RL indicates that the adsorption is favorable.
○ As far as our study is concerned, the obtained experimental results revealed that, for the different materials used for adsorption, Al cement displays great promise as an adsorbent, particularly due to its exceptional capacity to effectively remove fluoride from drinking water. In addition, it proves to be an interesting and worthwhile compound that deserves further as well as deeper investigation owing to its valuable characteristics and wide range of applications. From this perspective, this synthesis may be regarded as significant in terms of laying the ground for future researchers to extend this study further and explore fully and deeply the additional features of this compound.

## Figures and Tables

**Table 1:** Chemical compositions of synthesized samples.

Composite	MgO (g)	NH_4_H_2_PO_4_ (g)	Borax (g)	Al (g)	H_2_O (g)
S_1_	1.0790	2.8800	0.3310	0.2560	2
S_2_	1.0075	2.8250	0.3230	0.5620	2
S_3_	1.0680	2.8640	0.3115	1.5320	2

**Table 2:** BET surface, pore volume, and pore size of S1, S2, and S3.

Composite	BET Surface (m²/g)	Pore Volume (cm³/g)	Pore Size (Å)
S_1_	83.52	0.074	35.58
S_2_	52.27	0.055	41.24
S_3_	64.03	0.060	37.79

**Table 3:** Parameters of Langmuir, Freundlich, Temkin, and Dubinin–Radushkevich isotherm models.

Model	Sample 1	Sample 2	Sample 3
Langmuir	b = 0.205 L/g q_0_ = 4.87 mg/g R_l_ = 0.806 K_ap_ = −0.012 R^2^ = 0.999	b = 0.023 L/g q_0_ = 43.47 mg/g R_l_ = 0.993 K_ap_ = −0.000310 R^2^ = 0.999	b = −0.059 L/g q_0_ = −16.94 mg/g R_l_ = 0.988 K_ap_ = −0.0006 R^2^ = 0.997
Freundlich	1/n = 0.907 K_f_ = 0.007 R^2^ = 0.999	1/n = 1.022 K_f_ = 0.011 R^2^ = 0.997	1/n = 1.082 K_f_ = 0.0084 R^2^ = 0.962
Temkin	A = 0.052 B = 0.492 R^2^ = 0.894	A = 0.048 B = 1.304 R^2^ = 0.782	A = 0.172 B = 0.782 R^2^ = 0.675
Dubinin-Radushkevich	q_d_ = 0.951 B=119.4 R^2^ = 0.829	q_d_ = 1.799 B= 103.7 R^2^ = 0.796	q_d_ = 1.799 B= 126.3 R^2^ = 0.792

**Table 4:** Comparative fluoride removal capacity of some adsorbents.

Adsorbent	Removal Capacity (mg/g)	Reference
Activated quartz	1.16	Fan et al. (2003) [31]
Alumina cement	1.61	Sana et al. (2023) [4]
Zeolite cement	1.76	Sana et al. (2023) [4]
Porous granular ceramic	1.79	Nan Chen et al.(2010) [27]
H_2_O_2_ cement	2.21	Sana et al. (2023) [4]
Composite S_1_	2.35	Present study
Composite S_2_	4.51	Present study
Composite S_3_	4.84	Present study

## Data Availability

Data will be requested to the authors.
